# Dual effect of lithium on NFAT5 activity in kidney cells

**DOI:** 10.3389/fphys.2015.00264

**Published:** 2015-09-24

**Authors:** Christoph Küper, Franz-Xaver Beck, Wolfgang Neuhofer

**Affiliations:** ^1^Department of Physiology, University of MunichMunich, Germany; ^2^Medical Clinic V, University Hospital Mannheim, University of HeidelbergMannheim, Germany

**Keywords:** NFAT5, lithium, nephrogenic diabetes insipidus, GSK3β, urinary concentrating mechanism, chronic kidney disease

## Abstract

Lithium salts are used widely for treatment of bipolar and other mental disorders. Lithium therapy is accompanied frequently by renal side effects, such as nephrogenic diabetes insipidus or chronic kidney disease (CKD), but the molecular mechanisms underlying these effects are still poorly understood. In the present study we examined the effect of lithium on the activity of the osmosensitive transcriptional activator nuclear factor of activated T cells 5 (NFAT5, also known as TonEBP), which plays a key role in renal cellular osmoprotection and urinary concentrating ability. Interestingly, we found different effects of lithium on NFAT5 activity, depending on medium osmolality and incubation time. When cells were exposed to lithium for a relative short period (24 h), NFAT5 activity was significantly increased, especially under isosmotic conditions, resulting in an enhanced expression of the NFAT5 target gene heat shock protein 70 (HSP70). Further analysis revealed that the increase of NFAT5 activity depended primarily on an enhanced activity of the c-terminal transactivation domain (TAD), while NFAT5 protein abundance was largely unaffected. Enhanced activity of the TAD is probably mediated by lithium-induced inhibitory phosphorylation of glycogen synthase kinase 3β (GSK-3β), which is in accordance with previous studies. When cells were exposed to lithium for a longer period (96 h), cellular NFAT5 activity and subsequently expression of HSP70 significantly decreased under hyperosmotic conditions, due to diminished NFAT5 protein abundance, also resulting from GSK-3β inhibition. Taken together, our results provide evidence that lithium has opposing effects on NFAT5 activity, depending on environmental osmolality and exposure duration. The potential impacts of these observations on the diverse effects of lithium on kidney function are discussed.

## Introduction

The beginning of the widespread use of lithium as a mood stabilizer in the treatment of bipolar disorders in the early 1970s also saw the first reports of adverse renal side effects accompanying lithium treatment. The most common adverse effect is nephrogenic diabetes insipidus (NDI), due to a decreased expression of the aquaporin-2 (AQP-2) water channel and incorrect trafficking of AQP-2 to the luminal membrane in collecting duct cells (Marples et al., 1995; Kwon et al., 2000). Lithium-induced NDI, affecting up to 40% of all patients, can be detected within a few weeks after beginning lithium administration (Grünfeld and Rossier, 2009). Additionally, patients receiving lithium for more than 10 years have a significantly increased risk of developing chronic kidney disease (CKD; Bendz et al., 2010).

Lithium affects multiple molecular targets and signaling pathways, making it difficult to characterize the exact mechanisms by which the positive effects on the mood of psychiatric patients or the renal side effects are induced. In the last two decades, however, it has become increasingly evident that inhibition of the glycogen synthase kinase 3β (GSK-3β; Klein and Melton, 1996; Stambolic et al., 1996) plays a crucial role for the effects of lithium both in neuronal and kidney cells. GSK-3β is a ubiquitiously expressed serine/threonine kinase with multiple downstream targets, often transcriptional regulators (Doble and Woodgett, 2003). Under “normal” (non-stimulated) conditions GSK-3β is in its active state. In most cases, phosphorylation by active GSK-3β suppresses the activity of its downstream targets. Under stimulated conditions, phosphorylation of a serine residue (Ser9 in mouse, Ser21 in human) inactivates GSK-3β, thereby activating downstream targets.

Recently, the osmosensitive transcription factor nuclear factor of activated T-cells 5 [NFAT5, also known as tonicity enhancer binding protein (TonEBP) or osmolality response element binding protein (OREBP)] has been identified as GSK-3β downstream target in renal cells (Zhou et al., 2013; Quadri and Siragy, 2014). NFAT5 is an osmosensitive transcription factor that serves two important functions in the renal medulla. First, it regulates the expression of osmoprotective genes, such as heat shock protein 70 (HSP70; Woo et al., 2002), aldose reductase (AR; Ko et al., 2000), taurine transporter (TauT; Zhang et al., 2003), sodium-myo-inositol transporter (SMIT; Miyakawa et al., 1999), or betain-gaba transporter 1 (BGT-1; Miyakawa et al., 1998), all of which are essential for cell survival under the hyperosmotic conditions of the renal medulla. Second, NFAT5 (together with other factors) regulates the expression of components of the urinary concentration machinery, such as AQP-2 (Lam et al., 2004; Hasler et al., 2006), or the urea transporter-1 (UT-A; Nakayama et al., 2000). Accordingly, animal studies have demonstrated that inhibition or downregulation of NFAT5 in the kidney is associated with urinary concentration defects (Lam et al., 2004) and severe renal damages (López-Rodríguez et al., 2004). Generally, activation of NFAT5 in response to hyperosmotic stress is mediated by an increase of NFAT5 protein abundance (Miyakawa et al., 1999) and by activation of the c-terminal transactivation domain (TAD; Ferraris et al., 2002), which in turn stimulates the transcriptional machinery. The regulation of NFAT5 activity is a complex process, and various kinases, in addition to GSK-3β, are reportedly involved, among them p38 (Ko et al., 2002), AKT (Roth et al., 2010), or phosphoinositide-3 kinase (PI-3K; Irarrazabal et al., 2006). Under isosmotic conditions GSK-3β is active and suppresses NFAT5 activation (the exact phosphorylation site within NFAT5 has not yet been identified). High osmolality activates AKT, PKA, and PI-3K, which in turn inhibit GSK-3β, resulting in enhanced activity of the c-terminal TAD of NFAT5 (Zhou et al., 2013).

The aim of the present study was to evaluate the effects of lithium on the activity of NFAT5. We present evidence for two opposing effects: short exposure enhances NFAT5 activity by stimulation of the c-terminal TAD, while long exposure decreases NFAT5 activity due to diminished protein abundance.

## Methods

### Materials

Antibodies were obtained as follows: anti-NFAT5 antibody was from Santa Cruz Biotechnology (Santa Cruz, CA, USA); anti-actin antibody was from Sigma (Deisenhofen, Germany); anti-phospho-GSK-3β (Ser9), anti-GSK-3β, anti-phospho-p38 (Thr180/Tyr182), and anti-p38 were purchased from Cell Signaling (Beverly, MA, USA); anti-phospho-Akt (Ser473) and anti-AKT were from Genscript (Piscataway, NJ, USA). GSK-3β inhibitor VIII was obtained from Cayman Chemical (Ann Arbor, MI, USA). Unless otherwise indicated, other reagents were purchased from Biomol (Hamburg, Germany), Biozol (Eching, Germany), Carl Roth (Karlsruhe, Germany), or Sigma.

### Cell culture

HEK293 or IMCD-3 cells were cultured in Dulbeccos modified Eagles medium supplemented with 10% fetal bovine serum (Biochrom, Berlin, Germany), 100 units/ml penicillin, and 100 μg/ml streptomycin (Invitrogen, Karlsruhe, Germany) at 37°C in a humidified atmosphere (95% air/5% CO_2_). Cells were grown in 24-well plates to confluency. For experiments, medium osmolality was increased by addition of NaCl. Lithium was added to the medium as Li_2_SO_4_, in concentrations of 1–10 mM; control cells were treated with corresponding concentrations of Na_2_SO_4_. GSK-3β inhibitor VIII, dissolved in DMSO, was used at a final concentration of 10 μM.

### qRT-PCR analysis

For determination of mRNA expression levels, total RNA from IMCD-3 cells was recovered using TriFast Reagent (Peqlab, Erlangen, Germany) according to the manufacturer's recommendations. The primers (Metabion, Martinsried, Germany) used in these experiments were:

HSP70_fw: 5′-tga gtc cca cac tct cac ca-3′;
HSP70_rev: 5′-ctg tgg gtg aag ctg tta agg -3′;
NFAT5_fw: 5′-AAT CGC CCA AGT CCC TCT AC-3′;
NFAT5_rev: 5′-GGT GGT AAA GGA GCT GCA AG -3′;
actin_fw: 5′- CCA ACC GCG AGA AGA TGA-3′;
actin_rev: 5′- CCA GAG GCG TAC AGG GAT AG -3′.

Experiments were performed on a CFX Connect Real Time PCR Detection System (BioRad, Hercules, CA, USA) using the SensiMix SYBR One-Step Kit (Bioline, Luckenwalde, Germany) according to the manufacturer's recommendations. Relative mRNA expression of the respective genes was calculated by the 2^−ΔΔ*CT*^–method (Livak and Schmittgen, 2001), using β-actin as housekeeping gene. Specificity of PCR product formation was confirmed by monitoring melting point analysis and by agarose gel electrophoresis.

### Immunoblot analysis

Aliquots of cell extracts from IMCD-3 cells (5–30 μg protein) were subjected to sodium dodecylsulphate polyacrylamide gel electrophoresis (SDS-PAGE) and blotted onto nitrocellulose membranes (GE Healthcare, Pittsburgh, PA, USA). Non-specific binding sites were blocked with 5% non-fat dry milk in phosphate-buffered saline (PBS) containing 0.1% Tween-20 (PBS-T) at room temperature for 1 h. Samples were incubated with primary antibodies in PBS-T containing 5% non-fat dry milk over night at 4°C. Subsequently, the blots were washed three times with PBS-T for 5 min each, and the membranes incubated with appropriate secondary antibody at room temperature for 1 h in PBS-T containing 5% non-fat dry milk. After washing with PBS-T three times for 5 min each, immunocomplexes were visualized by enhanced chemiluminescence.

### Determination of NFAT5 cellular activity

NFAT5 transcriptional activity was assessed using the secreted alkaline phosphatase system (SEAP) with a reporter construct in which the SEAP open reading frame is under control of two TonE sites. Generation of stably transfected HEK293-pSeap-TonE cells is described elsewhere (Neuhofer et al., 2007). After growing to confluency, the cells were treated as indicated and SEAP activity in the medium determined as described in detail elsewhere (Neuhofer et al., 2007). In experiments in which cells were exposed for 96 h to the respective stimulus, medium was changed after 72 h to ensure that SEAP activity in the medium reflects the actual NFAT5 cellular activity.

### Determination of NFAT5 transactivating activity

NFAT5 transactivating activity in HEK293 cells was determined using the Gal4 binary assay as described elsewhere (Ferraris et al., 2002; Küper et al., 2012). Gal4-TonEBP-TAD contains the yeast Gal4 DNA binding domain fused in-frame to the TAD of NFAT5 [amino acids 548-1531; kindly provided by Dr. J. Ferraris (NIH, Bethesda, MD, USA)]. pFR-SEAP (Agilent, Santa Clara, CA, USA) contains five tandem repeats of the Gal4 binding site upstream of a minimal promoter and the SEAP ORF. Briefly, 4 × 10^6^ cells were transfected by electroporation with 20 μg pGal4-TonEBP-TAD and 20 μg pFR-SEAP (350 V, 950 μF; 4 mm cuvette) using a Genepulser Xcell apparatus (Bio-Rad), and subsequently plated into 4–6 wells of a 24-well plate. After 24–48 h, the cells were treated as indicated and SEAP activity in the medium was determined as described above.

### Statistical analyses

Data are expressed as means ± S.E.M. The significance of differences between the means was assessed by Student's *t*-test. *P* < 0.05 was regarded as significant. All experiments were performed at least four times and representative results are shown.

## Results

### Short-term exposure to lithium

First, the effect of a short-term exposure to lithium on NFAT5 activity in renal cells was tested. For this purpose, IMCD-3 or HEK 293 cells were exposed to 10 mM Li_2_SO_4_ or 10 mM Na_2_SO_4_ (as control) for periods of 24 h, under isosmotic (300 mosm/kg H_2_O) or hyperosmotic (500 mosm/kg H_2_O) conditions.

### Short-term exposure to lithium increases cellular NFAT5 activity

**Figure 1:**
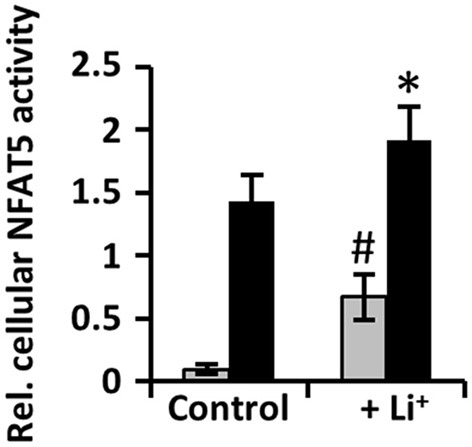
**Short-term exposure to lithium increases cellular NFAT5 activity**. HEK 293 cells stably transfected with a reporter construct in which the SEAP gene is under control of two TonE sites were incubated for 24 h in isosmotic (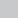; 300 mosm/kg H_2_O) or hyperosmotic (■; 500 mosm/kg H_2_O) medium and exposed to 10 mM Li_2_SO_4_ or 10 mM Na_2_SO_4_ (as control). After 24 h, SEAP activity was measured as described in Methods. Data are means ± SEM for *n* = 6; ^*^P < 0.05 vs. hyperosmotic control; ^#^*P* < 0.05 vs. isosmotic control.

Cellular NFAT5 activity was measured in HEK 293 cells stably transfected with a TonE-driven reporter vector. Under isosmotic conditions a four-fold increase of reporter gene activity in response to lithium was observed, while under hypertonic conditions lithium caused a more moderate increase of reporter gene activity about 25% (Figure 1). These data clearly indicate that lithium, especially under isosmotic conditions, stimulates basal cellular NFAT5 activity, but also slightly increases NFAT5 activity under hyperosmotic conditions.

### Lithium increases expression of the NFAT5 target gene HSP70

**Figure 2:**
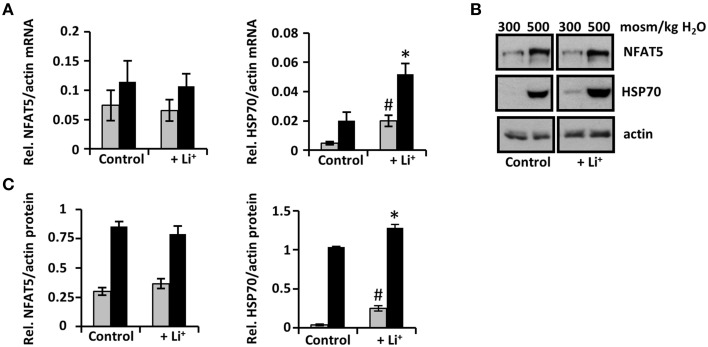
**Short-term exposure to lithium increases expression of the NFAT5 target gene HSP70**. Confluent IMCD-3 cells were incubated for 24 h in isosmotic (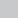; 300 mosm/kg H_2_O) or hyperosmotic (■; 500 mosm/kg H_2_O) medium and exposed to 10 mM Li_2_SO_4_ or 10 mM Na_2_SO_4_ (as control). **(A)** Cells were processed for RNA extraction and the abundance of NFAT5 and HSP70 mRNA transcripts determined by qRT-PCR. Relative mRNA abundance was normalized to that of β-actin to correct for differences in RNA input. Data are means ± SEM for *n* = 4; ^*^P < 0.05 vs. hyperosmotic control; ^#^*P* < 0.05 vs. isosmotic control. **(B)** Cells were processed for immunoblotting. To demonstrate comparable protein loading, the blots were also probed for actin. A representative blot from four independent experiments is shown. **(C)** Relative protein abundance of NFAT5 and HSP70 was quantified by densitometric analysis of immunoblots and normalized to that of actin to correct for differences in protein loading. Data are means ± SEM for *n* = 4; ^*^P < 0.05 vs. hyperosmotic control; ^#^*P* < 0.05 vs. isosmotic control.

To test the effect of increased cellular activity of NFAT5 on expression of NFAT5 target genes, mRNA and protein levels of HSP70 (as a representative NFAT5 target gene) were evaluated in IMCD-3 cells (Figures 2A–C). Expression of HSP70 under isosmotic conditions was enhanced severalfold compared with control cells. Under hyperosmotic conditions, lithium also increased expression of HSP70 (although the increase was less pronounced than under isosmotic conditions), consistent with the results of lithium-induced enhanced cellular NFAT5 activity.

### Cellular NFAT5 activity is increased by enhanced activation of the TAD

**Figure 3:**
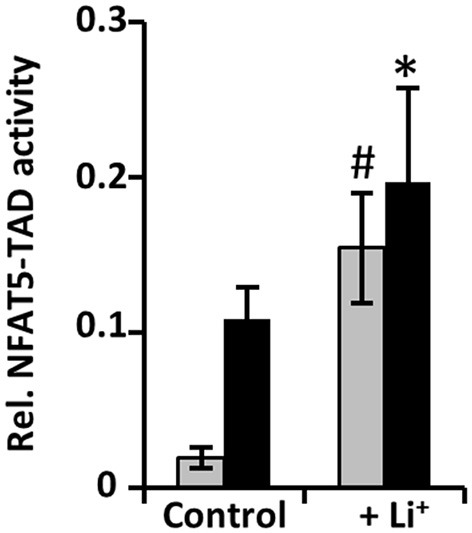
**Short-term exposure to lithium stimulates the transactivation domain of NFAT5**. HEK 293 cells were electroporated with pGAL4-TonEBP-TAD and pFR-SEAP as described in Methods. Subsequently, cells were incubated for 24 h in isosmotic (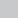; 300 mosm/kg H_2_O) or hyperosmotic (■; 500 mosm/kg H_2_O) medium and exposed to 10 mM Li_2_SO_4_ or 10 mM Na_2_SO_4_ (as control). Thereafter, SEAP activity was determined as described in Methods. Data are means ± SEM for *n* = 6; ^*^P < 0.05 vs. hyperosmotic control; ^#^*P* < 0.05 vs. isosmotic control.

To determine the mechanism, by which lithium increases NFAT5 cellular activity, NFAT5 expression and TAD activity were assessed. NFAT5 expression was assessed in IMCD-3 cells at the mRNA and protein levels. As shown in Figure 2, hyperosmolality increased NFAT5 expression, as expected, but lithium had no significant influence on NFAT5 mRNA or protein levels. The activity of the C-terminal TAD of NFAT5 was measured in HEK 293 cells, transiently transfected with a binary reporter system. As shown in Figure 3, lithium increased basal TAD activity approximately three-fold under isosmotic conditions, compared with control cells. Also under hyperosmotic conditions, lithium also significantly increased TAD activity. These results clearly indicate that an enhanced transactivating activity is by far the most important mechanism for lithium-induced increase of cellular NFAT5 activity.

### Lithium mediates inhibitory phosphorylation of GSK-3β

**Figure 4:**
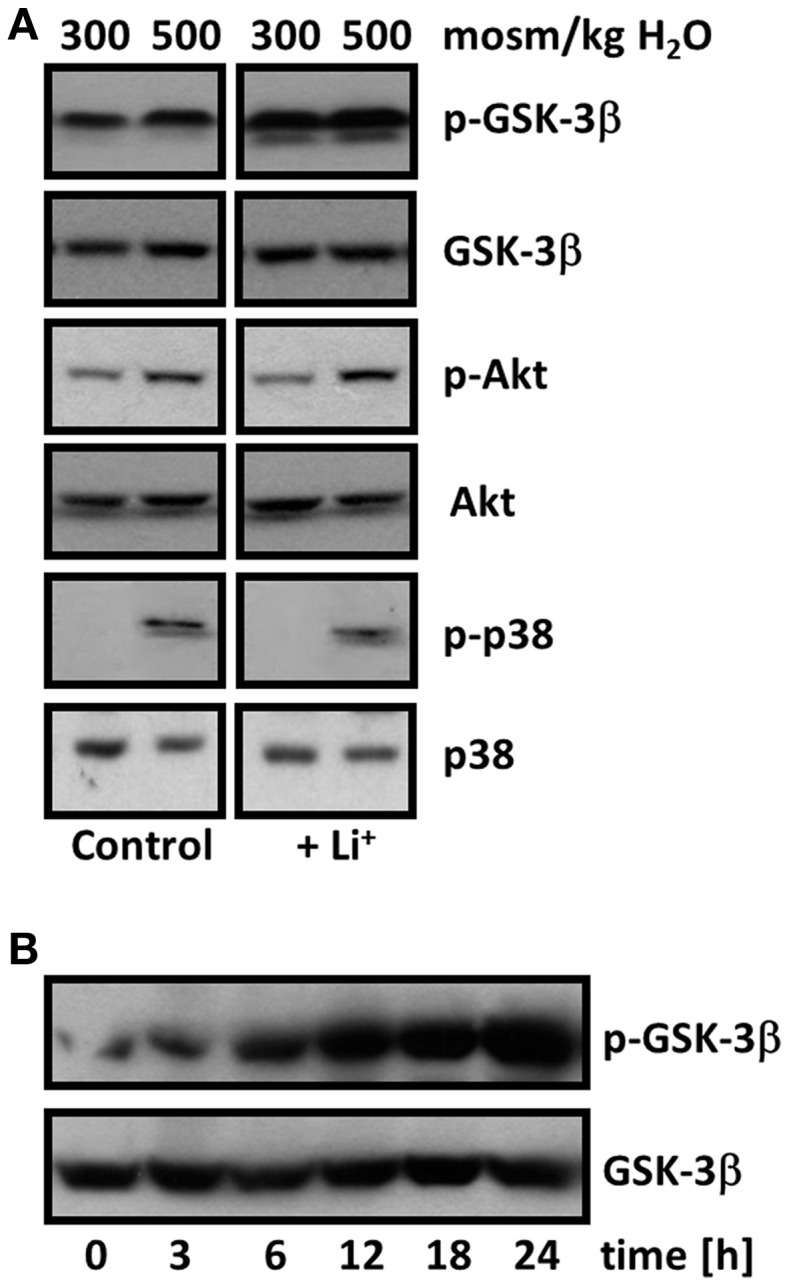
**Lithium mediates inhibitory phosphorylation of GSK-3β. (A)** Confluent IMCD-3 cells were incubated for 24 h in isosmotic (300 mosm/kg H_2_O) or hyperosmotic (500 mosm/kg H_2_O) medium and exposed to 10 mM Li_2_SO_4_ or 10 mM Na_2_SO_4_ (as control). Thereafter, cells were processed for immunoblotting and phosphorylation status of GSK-3β, AKT, and p38 was determined as described in Methods. A representative blot from four independent experiments is shown. **(B)** Time-course of GSK-3β inhibitory phosphorylation. Confluent IMCD-3 cells were incubated for 0–24 h in isosmotic medium (300 mosm/kg H_2_O) in the presence of 10 mM Li_2_SO_4_. Thereafter, cells were processed for immunoblotting and phosphorylation status of GSK-3β was determined as described in Methods. A representative blot from four independent experiments is shown.

Next, we examined the effect of lithium on GSK-3β in IMCD-3 cells. Lithium induced an increase of inhibitory phosphorylation at Ser9, as shown in Figure 4A. We also tested phosphorylation status of the kinases AKT and p38, which have been previously shown to mediate inhibition of GSK-3β in renal cells under hyperosmotic conditions. We could not detect any substantial impact of lithium on AKT or p38 activity, under either iso- or hyper-osmotic conditions. Time-course analysis revealed a relatively slow lithium-induced inhibitory phosphorylation of GSK-3β, which reached its maximum after approximately 24 h (Figure 4B).

### Long-term exposure to lithium

Additionally, the effect of prolonged exposure to lithium on NFAT5 activity in renal cells was examined. For this purpose, IMCD-3 or HEK 293 cells were again exposed to 10 mM Li_2_SO_4_ or 10 mM Na_2_SO_4_ (as control) under isosmotic (300 mosm/kg H_2_O) or hyperosmotic (500 mosm/kg H_2_O) conditions, this time for periods of 48–96 h.

### Long-term exposure to lithium decreases cellular NFAT5 activity under hyperosmotic conditions

**Figure 5:**
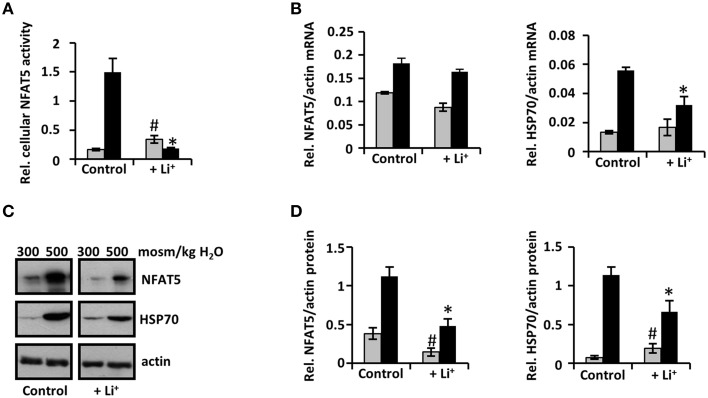
**Long-term exposure to lithium decreases cellular NFAT5 activity**. HEK 293 cells stably transfected with a reporter construct in which the SEAP gene is under control of two TonE sites **(A)** or IMCD-3 cells **(B–D)** were incubated for 96 h in isosmotic (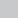; 300 mosm/kg H_2_O) or hyperosmotic (■; 500 mosm/kg H_2_O) medium and exposed to 10 mM Li_2_SO_4_ or 10 mM Na_2_SO_4_ (as control). **(A)** After 96 h, SEAP activity was measured as described in Methods. Data are means ± SEM for *n* = 6; ^*^P < 0.05 vs. hyperosmotic control; ^#^*P* < 0.05 vs. isosmotic control. **(B)** IMCD-3 cells were processed for RNA extraction and the abundance of NFAT5 and HSP70 mRNA transcripts was determined by qRT-PCR. Relative mRNA abundance was normalized to that of β-actin to correct for differences in RNA input. Data are means ± SEM for *n* = 4; ^*^P < 0.05 vs. hyperosmotic control; ^#^*P* < 0.05 vs. isosmotic control. **(C)** IMCD-3 cells were processed for immunoblotting. To demonstrate comparable protein loading, the blots were also probed for actin. A representative blot from four independent experiments is shown. **(D)** Relative protein abundance of NFAT5 and HSP70 was quantified by densitometric analysis of immunoblots and normalized to that of actin to correct for differences in protein loading. Data are means ± SEM for *n* = 4; ^*^P < 0.05 vs. hyperosmotic control; ^#^*P* < 0.05 vs. isosmotic control.

HEK 293 cells stably transfected with a TonE-driven reporter vector were incubated for 96 h. Whilst still slightly enhanced under isosmotic conditions, reporter gene activity in response to lithium decreased dramatically under hyperosmotic conditions, compared with control cells (Figure 5A). These data clearly indicate that prolonged exposure to lithium suppresses cellular NFAT5 activity under hyperosmotic conditions.

### Long-term exposure to lithium decreases expression of NFAT5 and HSP70

Next, expression of NFAT5 during long-term lithium exposure was assessed in IMCD-3 cells. While mRNA levels showed no significant differences compared to control cells (Figure 5B), NFAT5 protein abundance declined significantly after 48 h, especially under hyperosmotic conditions, but also under isosmotic conditions; this decline was further enhanced after 96 h (Figures 5C,D). These results indicate that long-term exposure to lithium decreases NFAT5 expression and thereby cellular NFAT5 activity, probably mediated by posttranscriptional mechanisms.

Expression of the NFAT5 target gene HSP70 under isosmotic conditions was slightly enhanced compared with control cells, even after 96 h. Under hyperosmotic conditions, HSP70 protein levels were significantly decreased after 96 h of exposure to lithium (Figures 5C,D). These results are consistent with the observed attenuation of cellular NFAT5 activity.

GSK-3β inhibitory phosphorylation by lithium was still stable after 96 h (data not shown).

### Pharmacological GSK-3β inhibition mimics the effect of lithium on NFAT5 activity

**Figure 6:**
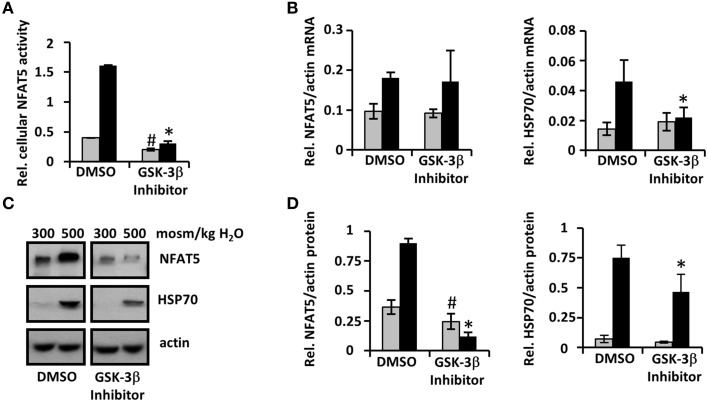
**GSK-3β inhibitor VIII mimics the effect of lithium on cellular NFAT5 activity**. HEK 293 cells stably transfected with a reporter construct in which the SEAP gene is under control of two TonE sites **(A)** or IMCD-3 cells **(B–D)** were incubated for 96 h in isosmotic (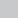; 300 mosm/kg H_2_O) or hyperosmotic (■; 500 mosm/kg H_2_O) medium in the presence of 10 μM GSK-3β inhibitor VIII or vehicle DMSO (as control). **(A)** After 96 h, SEAP activity was measured as described in Methods. Data are means ± SEM for *n* = 6; ^*^P < 0.05 vs. hyperosmotic control; ^#^*P* < 0.05 vs. isosmotic control. **(B)** IMCD-3 cells were processed for RNA extraction and the abundance of NFAT5 and HSP70 mRNA transcripts was determined by qRT-PCR. Relative mRNA abundance was normalized to that of β-actin to correct for differences in RNA input. Data are means ± SEM for *n* = 4; ^*^P < 0.05 vs. hyperosmotic control; ^#^*P* < 0.05 vs. isosmotic control. **(C)** IMCD-3 cells were processed for immunoblotting. To demonstrate comparable protein loading, the blots were also probed for actin. A representative blot from four independent experiments is shown. **(D)** Relative protein abundance of NFAT5 and HSP70 was quantified by densitometric analysis of immunoblots and normalized to that of actin to correct for differences in protein loading. Data are means ± SEM for *n* = 4; ^*^P < 0.05 vs. hyperosmotic control; ^#^*P* < 0.05 vs. isosmotic control.

To determine whether lithium-induced downregulation of NFAT5 is mediated by GSK-3β inhibition, we also examined the effect of the specific pharmacological GSK-3β inhibitor VIII. Similar to lithium treatment, under hyperosmotic conditions both cellular NFAT5 activity (Figure 6A) and expression of NFAT5 and HSP70 (Figures 6B–D) were decreased significantly compared with control cells. Under isosmotic conditions, cellular NFAT5 activity and HSP70 expression in cells treated with GSK-3β inhibitor VIII were also less than in control cells, in contrast to lithium treatment. We assume that an even stronger downregulation of NFAT5 by GSK-3β inhibitor VIII, compared with lithium, is responsible for this observation. Taken together, the results suggest that lithium-induced downregulation of NFAT5 might be mediated by GSK-3β inhibition.

## Discussion

The results presented in this study indicate that lithium has two opposing effects on NFAT5 activity in renal cells: in the first, “rapid” response to lithium exposure, NFAT5 activity increases. This increase is probably mediated by inhibition of GSK-3β and subsequent activation of the c-terminal TAD of NFAT5, while NFAT5 protein levels are largely unaffected. The group of Joan Ferraris showed recently that the TAD activity under isosmotic conditions is suppressed by GSK-3β, and that under hyperosmotic conditions, inhibitory phosphorylation of GSK-3β (mediated by AKT, PKA, and PI-3K) interrupts this suppression (Zhou et al., 2013). The lithium-induced increase of NFAT5 activity was especially pronounced under isosmotic conditions. Normally, NFAT5 activity under isosmotic conditions is virtually zero, as illustrated by the minimal expression of the NFAT5 target gene HSP70. Under these conditions, lithium increases NFAT5 activity and expression of HSP70 severalfold (however, still less than under hyperosmotic conditions). Under hyperosmotic conditions, the effect of lithium on NFAT5 activity is more moderate, probably since GSK-3β is already inhibited under these conditions, as mentioned above. An additional GSK-3β inhibition by lithium only slightly enhances TAD activity (25–50%).

When cells were exposed to lithium for prolonged periods (up to 96 h), a second, “slow” response was observed: under hyperosmotic conditions, cellular NFAT5 activity and expression of NFAT5 target genes decreased, probably due to reduced NFAT5 protein abundance. The mechanisms, by which lithium-induced inhibition of GSK-3β mediates NFAT5 downregulation, are not clear. Normally, NFAT5 protein abundance increases under hyperosmotic conditions, which, in addition to activation of TAD, contributes to enhanced cellular NFAT5 activity under these conditions. Enhanced transcription and increased mRNA stability have been identified as important mechanisms for hyperosmolality-induced upregulation of NFAT5 expression (Cai et al., 2005). Since NFAT5 mRNA levels were largely unaffected by prolonged exposure to lithium, we assume that translational or posttranslational mechanisms are probably responsible for the observed downregulation of NFAT5. Regulation of NFAT5 by such mechanisms has, to date, not been addressed in detail. In cardiac cells, doxorubicin induces ubiquitin-independent proteasomal degradation of NFAT5 protein (Ito et al., 2007), and recent investigations suggest that the long non-coding RNA “non-coding repressor of NFAT” (NRON) may be involved in proteasomal NFAT5 degradation (Umekita et al., 2013). We attempted to establish whether the proteasomal inhibitor MG-132 influences lithium-induced degradation of NFAT5, but since exposure of IMCD-3 cells to the combination of hyperosmotic stress, lithium and MG-132 for periods longer than 24 h caused massive cell death, we were not able to obtain significant results (data not shown).

Of particular interest is the observation that the impact of prolonged lithium incubation (96 h) on NFAT5 activity also depends on environmental osmolality. As discussed above, NFAT5 activity and expression of target gene HSP70 clearly declines under hyperosmotic conditions. Under isosmotic conditions, however, NFAT5 activity in lithium treated cells is higher than in control cells, although NFAT5 protein abundance slightly decreased. We assume that, in sum, the strong stimulatory effect of lithium on TAD activity of NFAT5 overrides the decrease of NFAT5 protein, as also indicated by the increased expression of HSP70 under these conditions. Transferred to the situation in the kidney, these results indicate that the effect of lithium on NFAT5 activity depends not only on the duration of exposure but also on the kidney region. Cortical collecting duct cells are not usually exposed to hyperosmotic stress, hence, NFAT5 activity in this region may be enhanced even during long-term lithium treatment. In contrast, in collecting duct cells of the outer and especially the inner medulla, which are regularly exposed to hyperosmotic conditions, lithium probably decreases NFAT5 activity and expression of its target genes. Accordingly, a proteomic analysis demonstrated that expression of the NFAT5 target genes AR and HSP70 is significantly decreased in inner medullary collecting duct cells in lithium-treated rats (Nielsen et al., 2008).

The question arises as to whether altered NFAT5 activity contributes to the well-known renal side effects of lithium therapy. The molecular mechanisms underlying these side effects are not completely understood (Behl et al., 2015). Among these side effects, NDI is the most frequent, affecting up to 40% of lithium-treated patients. Lithium decreases AQP-2 expression and trafficking to the luminal membrane of the principal cells of the collecting duct. Moreover, the urea transporters UT-A and UT-B, which are necessary for efficient urea circulation in the kidney and hence for maximal urinary concentration ability, are downregulated by lithium in a rat model (Klein et al., 2002; Blount et al., 2010). NFAT5 has been identified as a positive regulator of AQP-2 (Kasono et al., 2005; Hasler et al., 2006) and UT-A (Nakayama et al., 2000) expression; additionally, the NFAT5 target gene AR has been linked to the urinary concentration mechanism (Aida et al., 2000). Accordingly, in a mouse model expressing a dominant-negative NFAT5 derivative, the urinary concentrating ability is decreased (Lam et al., 2004). In a rat model of sepsis-induced polyuria, inhibitory nitrosylation of NFAT5 is associated with decreased renal expression of AQP-2, UT-A1, ClC-K1 and its regulatory subunit barttin, also resulting in impaired urinary concentration ability (Küper et al., 2012). These data clearly indicate the importance of NFAT5 for urinary concentration, and thus it seems plausible that lithium-induced downregulation of NFAT5, at least in medullary collecting duct cells, contributes to the development of NDI during lithium treatment.

Long-term treatment with lithium (>10 years) is also associated with an enhanced risk of developing CKD (Markowitz et al., 2000; Presne et al., 2003). The underlying mechanisms are even less well understood than lithium-induced NDI, thus it is difficult to hypothesize whether downregulation of NFAT5 contributes to the very slow development of CKD. Since NFAT5 is an essential regulator for expression of several important osmoprotective genes it is easily imaginable that decreased NFAT5 activity might cause renal injury. Indeed, NFAT5 knockout mice develop severe renal damage (López-Rodríguez et al., 2004). However, to our knowledge there are, to date, no reports in the literature that clearly link decreased expression of one or more NFAT5 target genes to lithium-induced CKD, thus this subject remains to be elucidated.

In contrast to these well-known adverse renal side effects of lithium, recent reports indicate that lithium might be useful for treatment of acute kidney injury (AKI). In animal models of ischemia/reperfusion- or toxin-induced AKI, lithium was protective against renal injury or improved recovery from AKI (Wang et al., 2009; Talab et al., 2010, 2012; Plotnikov et al., 2013; Bao et al., 2014). It has been shown before that upregulation of HSP70 can protect renal cells against ischemic injury (Aufricht et al., 1998; Yeh et al., 2010; Wang et al., 2011). Based on the results of the present study, one may speculate that short exposure to lithium increases NFAT5 activity in renal cells and thereby stimulates expression of HSP70 (and perhaps other target genes), which contribute to the protective effect of lithium against renal injury.

Can lithium alter cellular NFAT5 activity also in extrarenal tissues? When lithium is used therapeutically, serum levels of 0,4-1 mM are recommended (National Institute for Health and Care Excellence, (NICE), 2006). In the present study, significant effects of lithium on NFAT5 activity in renal cells were only observed at relative high concentrations (5–10 mM Li_2_SO_4_, corresponding to 10–20 mM Li^+^). Due to the concentration mechanism of the nephron, lithium levels in the collecting duct fluid can exceed 20 mM (Hayslett and Kashgarian, 1979; Birch and Hullin, 1980), indicating that the effects we observed in cell culture may also be relevant *in vivo*, at least in the distal nephron and the collecting duct. Based on our results, it seems unlikely that the comparatively low serum lithium levels will have a significant impact on NFAT5 activity in extrarenal cells. However, accumulation of lithium has also been observed in other tissues (Thomas et al., 1975; Mendlewicz et al., 1978; Costa et al., 1982; Kabakov et al., 1998), and thus lithium-mediated altered NFAT5 activity outside the kidney should not be completely ruled out.

### Conflict of interest statement

The authors declare that the research was conducted in the absence of any commercial or financial relationships that could be construed as a potential conflict of interest.
